# Development of a core outcome set for traumatic brachial plexus injury

**DOI:** 10.1177/17531934231212973

**Published:** 2023-11-21

**Authors:** Caroline Miller, Jane Cross, Dominic M. Power, Christina Jerosch-Herold, International brachial plexus consensus group, Amy Moore

**Affiliations:** 1 School of Health Sciences, University of East Anglia, Norwich, UK; 2 Therapy Services, Queen Elizabeth Hospital Birmingham, University Hospitals Birmingham Foundation Trust, Birmingham, UK; 3 The Peripheral Nerve Injury Service, University Hospitals Birmingham Foundation Trust, Birmingham, UK

**Keywords:** Nerve injury, outcome measures, upper limb, trauma, patient-reported outcomes

## Abstract

The aim of the present study was to reach international consensus on the minimum set of outcomes to measure and report in adult traumatic brachial plexus injury care and research. This would facilitate comparison of outcomes from different centres and meta-analysis in research. A list of outcomes was developed from a systematic review (*n* = 54) and patient interviews (*n* = 12). The outcomes were rated in a three-round online Delphi survey completed by international surgeons, patients and therapists. Two online consensus meetings with patients and clinicians ratified the final core outcome set. A total of 72 people (20 surgeons, 21 patients, 31 therapists) from 19 countries completed all survey rounds. Thirty-eight people from nine countries attended separate patient (*n* = 13) and clinician consensus (*n* = 25) meetings. Outcomes were included if recommended by more than 85% of contributors. Pain, voluntary movement and carrying out a daily routine are the core outcome domains that should be assessed and reported when treating and researching adults with a traumatic brachial plexus injury.

**Level of evidence:**

V

## Introduction

There is uncertainty over which treatments are most effective for individuals with a traumatic brachial plexus injury (TBPI). Inconsistency in outcome selection across adult TBPI studies and peripheral nerve centres (Dy et al., 2015; Miller et al., 2021) has limited the opportunity for data assimilation and meta-analysis (Ayhan et al., 2019; Donnelly et al., 2020). Furthermore, clinicians and researchers have to date largely focused on measuring impairment (Dy et al., 2015; Miller et al., 2021). Therefore, it is difficult to make recommendations for care and there remains a risk that clinicians and researchers measure outcomes that are not a priority for patients (Kirwan et al., 2003).

To reduce outcome heterogeneity, improve the ability to pool outcomes and ensure that the outcomes measured are important to all stakeholders, the COMBINE (Core Outcome Measures in Brachial plexus INjuriEs) project was undertaken to develop a core outcome set (COS) for routine practice and research in TBPI (COS-TBPI). A COS is an agreed minimum set of outcomes that should be measured and reported in a health condition (Williamson et al., 2012). They can be developed for the purposes of research, routine care or both (Gargon et al., 2021). A COS should have input from key stakeholders, including patients and healthcare professionals (Williamson et al., 2017).

Use of a COS increases consistency in outcome reporting, allowing more trials to be included in meta-analyses and ensuring that relevant outcomes are measured (Kirkham et al., 2017a). Selective reporting bias is also reduced since it becomes apparent if COS outcomes are not fully reported. In clinical practice, a COS for TBPI could facilitate collaboration between tertiary peripheral nerve centres using routine or audit data to create larger datasets to inform evidence. Use of a COS also supports monitoring of safety and quality of TBPI interventions, detecting interventions with poor outcomes at an early stage and preventing their widespread use.

## Methods

**Figure 1.**
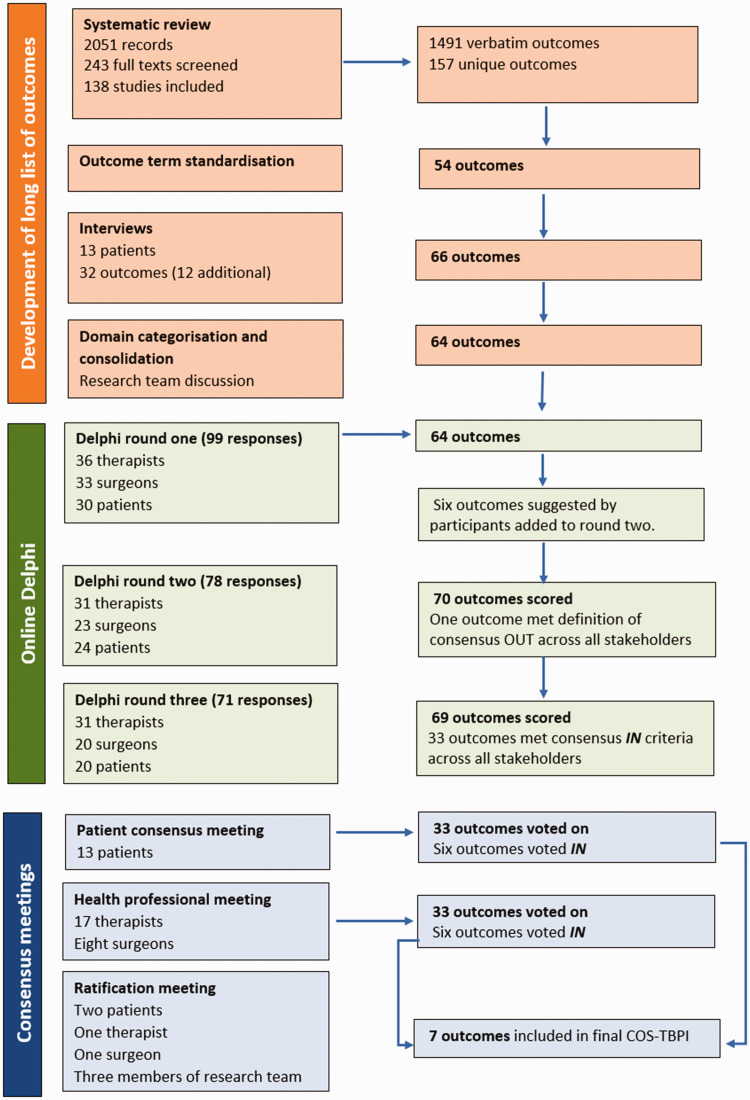
Overview of the COS-TBPI development process.

The COS-TBPI was developed in three phases (Figure 1): (1) generation of a longlist of outcomes; (2) a three-round online Delphi; and (3) consensus meetings to agree the final COS-TBPI. The study adhered to the minimum standards for design of a COS study (Core Outcome Set-Standards for Development [COS-STAD]) and included consideration of scope, stakeholders and consensus process (Kirkham et al., 2017b). The COMBINE project protocol has been previously published (Miller et al., 2019). The study was prospectively registered with the Core Outcome Measures in Effectiveness Trials (COMET) Initiative (COMET, 2022a).

### Phase 1: generation of the longlist of outcomes

The longlist of outcomes was generated from two sources: first, through a systematic review of outcomes reported in studies evaluating interventions for individuals with a TBPI (Miller et al., 2021); and second, through patient interviews to identify outcomes important to adults with a TBPI (Miller et al., 2022). The outcomes extracted from the systematic review and identified from the interviews were grouped into domains and categorized into a taxonomy developed to support COS generation (Dodd et al., 2018) by the lead author (CM) and reviewed by the co-authors. All outcomes were written in plain English, and feedback was sought from the research team, clinicians and patient advisers on the clarity and understanding of the wording.

### Phase 2: online Delphi consensus study

The list of outcomes was used to populate an online Delphi Survey, which was administered using DelphiManager (COMET, 2022b). Good practice guidelines (Kirkham et al., 2017b) for COS development report that, as a minimum, healthcare professionals (HCPs), potential users of the COS in research and patients should be included in COS development. To ensure that the COS-TBPI reflected outcomes important to all stakeholders, individuals with a TBPI, specialist surgeons, therapists, nurses, pain physicians, psychologists and other HCPs who cared for or conducted research with adults with a TBPI were eligible and invited to participate in the Delphi. Eligibility criteria and sample size are detailed further in the study protocol (Miller et al., 2019). Patients were recruited through TBPI Facebook community sites, national TBPI charities and online forums. A video was designed to promote the study in collaboration with the patient advisory group (https://www.youtube.com/watch?v=7k6MYpugvRk). Adults with TBPI contacted the lead author if they were interested in participating and eligibility was confirmed. HCPs were recruited by direct email, with a participant information sheet attached including a link to Delphi registration. This was sent to international authors (*n* = 22), lead clinicians in tertiary TBPI international centres (*n* = 24) and attendees of an international TBPI conference (NARAKAS, Leiden 2019), who had registered an interest in participating in the study and consented to communication (47 therapists and nurses and 52 surgeons). The NARAKAS group also promoted the study through its distribution list. Finally, a Twitter site was set up to promote the study.

The online Delphi consisted of three sequential rounds of questionnaires with the same group of participants. During Delphi registration, participants assigned themselves to one of three stakeholder groups: (1) individual with a TBPI; (2) surgeon; and (3) other HCPs. Separating stakeholder groups into different panels ensures that each group’s views are equally represented, irrespective of panel size. Furthermore, the authors hypothesized that the three stakeholder groups may have diverse opinions and were interested in understanding these differences. In each round, participants were presented with and asked to rate how important each outcome was on a scale of 1–9 labelled as: 1–3 = not important; 4–6 = important but not critical; and 7–9 = critically important (Guyatt et al., 2011). In round 1, participants had the option to add outcomes that they felt were missing. These were reviewed by the research team and any outcome not already represented was added to round 2. Regardless of the ratings from round 1, no outcomes were removed between rounds 1 and 2.

In round 2, participants were shown the score they gave each outcome in the previous round, together with the distribution of scores from other participants within their own stakeholder group. Participants were invited to reflect on the similarities and differences observed before proceeding to rescore each outcome.

In round 3, items from round 2 continued to round 3 if they were rated 7–9 (critically important) by 50% or more and 1–3 (not important) by less than 15% in any stakeholder group. Participants were shown the score they gave each outcome in the previous round, together with the distribution of scores from participants in their own and other stakeholder groups separately. Participants were invited to reflect on the information and rescore each outcome again.

### Protocol deviation

One change to the protocol (Miller et al., 2019) was made by the research team after analysis of round 3 of the Delphi. The original protocol stated that any outcome reaching consensus criteria (≥70% scoring an outcome of 7–9) in any stakeholder group would be discussed at the consensus meetings (Miller et al., 2019). However, at the end of the Delphi, many outcomes reached consensus and it would not have been feasible to take all of them to the consensus meetings. Therefore, more stringent criteria were agreed by the research team and applied for the selection of outcomes to carry through to the final consensus meetings. Instead of items reaching consensus criteria in any stakeholder group being taken forwards, items rated 7–9 by at least 70% of participants in all stakeholder groups were discussed at the consensus meeting as recommended by OMERACT (Outcome Measures in Rheumatology) in their updated guidelines (Beaton et al., 2021).

### Phase 3: consensus meetings

The Delphi results were presented at two separate online consensus meetings for patients and HCPs. Meetings were held virtually using ZOOM videoconferencing. Adults with a TBPI, who participated and completed all three Delphi rounds, were invited to attend the patient consensus meeting. Surgeons and therapists were purposively sampled, from those who completed all three Delphi rounds, to represent a wide geographical area and different professions. Members of the core research team (CJH, JC and DP) attended all meetings to support the process but did not vote.

Before the meeting, participants were emailed a word version of all outcomes, which had reached consensus criteria after round 3 (Table S1). Participants were asked to consider these outcomes and identify their top 10, as voting in the consensus meeting would be restricted to ensure they voted for outcomes they viewed as critical in clinical practice and research.

The meeting was chaired by the lead author (CM), who has a background in TBPI care and research. Outcomes that reached consensus after round 3 for all groups were presented on Microsoft PowerPoint. Participants discussed groups of outcomes and then voted ‘Yes’ (this outcome should be included in the COS) or ‘No’ (this outcome should not be included) using the anonymous polling system on ZOOM. Voting results (in percentage voted ‘in’ and voted ‘out’) for the group of outcomes were presented immediately after voting. Outcomes voted ‘in’ by 85% or more participants (Damhuis et al., 2020), were taken forwards to the final ratification meeting for inclusion in the COS-TBPI. Outcomes voted ‘in’ by 70%–84% of the participants were discussed and subject to a further vote. Outcomes receiving 69% or fewer votes were removed in keeping with other COS consensus meetings (McNair et al., 2016). Discussion and further rounds of voting were undertaken until a consensus was reached on all outcomes.

A final ratification meeting with patient and HCP representatives was held to discuss the results from both meetings and ratify a final feasible COS-TBPI. A therapist and two patient representatives who had completed the Delphi and attended the consensus meeting were invited to the ratification meeting. The surgeon (DP) invited was experienced in treating patients with a TBPI and was also part of the research team. JC and CJH also attended the meeting as part of the research team. The remit of the meeting was to ratify outcomes voted ‘in’ at both consensus meeting and discuss outcomes voted ‘in’ at patient-only or HCP-only meetings. CM presented a summary of outcomes voted in by only one stakeholder group. These were reviewed and discussed. Outcomes voted ‘in’ at both meetings were confirmed and scope discussed if needed. Consensus on the domains to be included in the final COS-TBPI was reached.

## Results

### Generation of the longlist of outcomes

From the systematic review (Miller et al., 2019), 157 unique outcomes were identified from 138 studies and classified into 54 standardized outcome domains. Interviews with 13 adults with a TBPI (Miller et al., 2022) identified 32 outcomes, including 12 not identified through the systematic review. Through discussion with clinical experts and the research team these 66 outcomes were converted into 64 items, which populated the online Delphi. The 64 items, with plain language descriptors, were grouped into 10 domains (physical signs, sensation and pain, neurophysiology and structure of the nervous system, activities of daily living, social well-being, emotional well-being, sleep and overall health, delivery of care, costs of care and complications) (Table S2).

### Online Delphi

In total, 99 participants (33 surgeons, 30 adults with a TBPI and 36 therapists) from 21 countries took part in round 1 of the online Delphi. Seventy-one participants scored all outcomes for rounds 1–3. Tables 1 and 2 present participant demographics. Participants added 68 comments regarding potential additional outcomes in round 1. From these suggestions, six new outcomes were added to round 2 and wording for seven existing outcomes revised (Table S3). At the end of round 3, 33 outcomes met the consensus criteria for inclusion (Table 3). The attrition rate for participants from round 1 to round 3 was 29% (71 of 99 participants remained) with the highest attrition for surgeons.

**Table 1.** Demographics of surgeons and therapists in rounds 1–3.

	Round 1 survey (*n* = 69)	Round 2 survey (*n* = 54)	Round 3 survey (*n* = 51)
Healthcare professional occupation			
Therapist	36 (52)	31 (57)	31 (61)
Surgeon	33 (48)	23 (43)	20 (39)
No. of new patients with TBPI seen per month			
≤1	13 (19)	10 (19)	9 (18)
2–3	13 (19)	10 (19)	9 (18)
4–5	16 (23)	13 (24)	12 (24)
6–10	10 (15)	8 (15)	8 (16)
>10	13 (19)	10 (19)	10 (20)
Not stated	4 (5.8)	3 (5.5)	3 (5.8)

Data are presented as *n* (%).

**Table 2.** Demographic information for adults with TBPI in Delphi rounds 1–3.

Characteristic	Round 1 survey (*n* = 30)	Round 2 survey (*n* = 24)	Round 3 survey (*n* = 20)
Sex			
Male	26 (87)	20 (83)	17 (85)
Female	4 (13)	4 (17)	3 (15)
Age (years)			
<30	3 (10)	3 (13)	2 (10)
31–50	17 (57)	12 (50)	11 (55)
≥51	10 (33)	9 (38)	7 (35)
Time since diagnosis			
<6 months	0 (0)	0 (0)	0 (0)
7–12 months	2 (6.6)	2 (8.3)	1 (5)
1–2 years	7 (23)	5 (21)	4 (20)
3–5 years	5 (17)	4 (17)	4 (20)
>5 years	15 (50)	12 (50)	10 (50)
No response	1 (3.3)	1 (4.2)	1 (5)
Had surgery			
Yes	27 (90)	23 (96)	19 (95)
No	3 (10)	1 (4.2)	1 (5)

Data are presented as *n* (%).

TBPI: traumatic brachial plexus injury.

**Table 3.** Results of patient consensus meeting (13 participants).

Outcomes	Include (*n*)	Exclude (*n*)	Revote – Include	Revote – Exclude	Final decision
Voluntary movement of the arm	13	0			**Include**
Strength of the arm	7	6			Exclude
Carrying and lifting	2	11			Exclude
Fine hand movement	7	5			Exclude
Ability to feel with the arm	4	9			Exclude
Ability to feel to protect the arm from injury	7	6			Exclude
Pain intensity	11	2			**Include**
Pain duration	9	4			Exclude
Pain description	3	10			Exclude
Overall health	6	7			Exclude
Access to treatment	10	3	13	0	**Merge and include**
Appropriateness of treatment	11	2			
The ability of the brachial plexus to send signals to the skin and muscles of the arm	7	6			Exclude
Carrying out daily routine	11	2			**Include**
Maintaining personal hygiene	6	7			Exclude
Putting on and taking off clothes	5	8			Exclude
Ability to eat using utensils /hands	5	8			Exclude
Effect on relationship with or ability to care for children	6	7			Exclude
Emotional distress	2	11			Exclude
Self-esteem and self-confidence	3	10			Exclude
Ability to cope	9	4			Exclude
Expectations of treatment	8	5			Exclude
Loss of voluntary movement	13	0			**Include**
Loss of assisted movement (passive)	4	9			Exclude
Limited voluntary movement because of inability to coordinate muscles at the same time	7	6			Exclude
Nerve forms a painful bundle of nerves (neuroma)	7	6			Exclude
Damage to other nerves during the surgery	7	6			Exclude
Worsening of existing pain or pins and needles	13	0			**Include**
Failure of a surgical join of the nerve	10	3	10	3	Unsure
Failure of a surgical join of an artery of a vein	4	9			Exclude
Injury to an artery or vein resulting in bleeding where the operation takes place	3	10			Exclude
Development of a blood clot	4	9			Exclude
Breathing problems	7	6			Exclude

### Consensus meetings

In total, 38 participants from nine countries attended two consensus meetings (25 clinicians and 13 individuals with a TBPI) (Table S4).

After the initial round of voting in the patient consensus meeting, six outcomes were voted ‘in’ (voluntary movement of arm, pain intensity, appropriateness of treatment, carrying out daily routine and complications: loss of voluntary movement and worsening of existing pain or pins and needles) and 25 were voted ‘out’ (Table 3). Two items (‘access to treatment’ and ‘the complication failure of a surgical join of a nerve’) were discussed and revoted on. After the revote, ‘access to treatment’ was voted ‘in’ and no consensus was reached on ‘failure of a surgical join of the nerve’. The research team decided to carry forward this item (‘failure of a surgical join of the nerve’) to the ratification meeting. After voting, there was discussion about two of the items: ‘access to treatment’ and ‘appropriateness of treatment’ and patients decided to merge these items into one called ‘access to appropriate treatment’.

After voting at the HCP consensus meeting, five outcomes were voted ‘in’ (Table 4). This included voluntary movement, fine hand movement, ability to feel with the arm, carrying out daily routine, surgical complications: loss of voluntary movement (donor or affected limb). The three pain outcomes (pain intensity, duration and frequency, and description) did not reach consensus. Participants expressed concern about this and felt that the votes had been split between the separate pain outcomes and considered it important to be part of the COS. Participants asked and agreed to revote on pain as a whole (including pain intensity, duration and frequency, and description). It reached the consensus criteria. In total, HCPs voted ‘in’ six outcomes (Tables 4 and 5).

**Table 4.** Results of health professional consensus meeting (25 participants).

Outcomes	Include (*n*)	Exclude (*n*)	Revote – include	Revote – exclude	Final decision
Voluntary movement of the arm (*n* = 24)	19	5	23	2	**Include**
Strength of the arm (*n* = 24)	15	9			Exclude
Carrying and lifting (*n* = 24)	11	13			Exclude
Fine hand movement (*n* = 24)	19	5	22	3	Merge**Include**
Ability to feel with the arm (*n* = 25)	18	7	24	1	
Ability to feel to protect the arm from injury (*n* = 25)	4	21			Exclude
Pain intensity (*n* = 25)	16	9			Exclude
Pain duration and frequency (*n* = 25)	5	20			Exclude
Pain description (quality and interference) (*n* = 25)	7	18			Exclude
Overall health	9	16			Exclude
Access to treatment	10	15			Exclude
Appropriateness of treatment	6	19			Exclude
The ability of the brachial plexus to send signals to the skin and muscles of the arm	7	18			Exclude
Carrying out daily routine (*n* = 24)	22	2			**Include**
Maintaining personal hygiene (*n* = 24)	6	18			Exclude
Putting on and taking off clothes (*n* = 24)	4	20			Exclude
Ability to eat using utensils /hands (*n* = 24)	7	17			Exclude
Effect on relationship with or ability to care for children (*n* = 25)	6	19			Exclude
Emotional distress	14	11			Exclude
Self-esteem and self-confidence	9	16			Exclude
Ability to cope	15	10			Exclude
Expectations of treatment	9	16			Exclude
Pain (combining intensity, frequency and duration, and description)	–	–	25		**Include**
Loss of voluntary movement	19	6	24	0	**Include**
Loss of assisted movement (passive)	11	14			Exclude
Limited voluntary movement because of inability to coordinate muscles at the same time	11	14			Exclude
Nerve forms a painful bundle of nerves (neuroma)	12	13			Exclude
Damage to other nerves during the surgery	15	10			Exclude
Worsening of existing pain or pins and needles	15	10			Exclude
Failure of a surgical join of the nerve (*n* = 24 then 25)	19	5	19	6	Unsure
Failure of a surgical join of an artery of a vein	6	18			Exclude
Injury to an artery or vein resulting in bleeding where the operation takes place	6	18			Exclude
Development of a blood clot	8	16			Exclude
Breathing problems	9	15			Exclude

**Table 5.** Comparison of outcomes reaching consensus at patient and HCP meeting.

Outcomes	Both patient and HCP meetings	Patient only	HCP only
Voluntary movement of the arm	X		
Carrying out daily routine	X		
Loss of voluntary movement (donor complication)	X		
Pain intensity		X	
Pain (including intensity, duration and frequency, and description)			X
Access to appropriate treatment		X	
Worsening of existing pain or pins and needles (donor complication)		X	
Fine hand movement			X
Ability to feel with the arm			X

HCP: healthcare professional.

One outcome, ‘failure of a surgical join of the nerve’, did not reach the criteria for inclusion (85% or more voting it ‘in’) or exclusion (69% or less voting it ‘in’), even with revoting. This outcome was carried forward to be discussed at the ratification meeting with patients and HCPs. There was concern at the end of the clinicians’ consensus meeting that none of the emotional well-being outcomes that had been on voted reached consensus. Therapists and surgeons emphasized that the emotional well-being outcomes significantly impacted on a patient’s recovery and that measuring them was important. There was agreement that the distribution of votes would be analysed by the research team and presented and reviewed at the final ratification meeting.

### Ratification meeting

The research team (CM, JC, CJH), two patient representatives, one therapist and one surgeon attended the online final ratification meeting on 20 April 2021. All outcomes voted ‘in’ at both meetings were included in the COS-TBPI. Attendees agreed to include fine hand movement within the ‘voluntary movement of the arm’ outcome. It was agreed appropriate to include a larger pain domain (including intensity, duration and description). Attendees discussed the merits of having several tiers, such as other COS (Tong et al., 2020), and as OMERACT recommends (Beaton et al., 2021). Tier 1 would include outcomes that all stakeholders agreed as important to include. Tier 2 would include outcomes that one stakeholder group agreed were critically important. Tier 1 outcomes would always be measured and reported in clinical practice and research, while Tier 2 outcomes are important but optional to measure.

Attendees discussed the outcomes within the emotional well-being domain. CM presented the distribution of votes across the different emotional well-being outcomes (emotional distress, self-esteem and confidence, expectations of treatment, ability to cope). At least one of the outcomes from the domain ‘emotional well-being’ had been selected for inclusion by every participant in the clinician meeting. However, the votes were split between multiple outcomes and no single one reached the required consensus threshold. At the patient meeting, 10 of 13 (76%) participants voted to include at least one emotional well-being outcome. Emotional distress and ability to cope were the highest-rated outcomes (in the ‘emotional well-being’ domain) in both meetings. After discussion, attendees at the ratification meeting agreed that emotional distress and ability to cope should therefore be included in Tier 2 of the COS-TBPI.

Finally, two surgical complications outcomes were discussed. Loss of voluntary movement was voted ‘in’ at both meetings. However, ‘worsening of existing pain or pins and needles’ was only voted ‘in’ by the patient group. There was general agreement that ‘worsening of existing pain or pins and needles’ would not be appropriate to fit into Tier 2 where it would be optional, as it was felt an important outcome in donor morbidity. In addition, there was discussion that having one complication in Tier 1 and another in Tier 2 may be confusing for future users of the COS. It was agreed therefore that these two surgical complication outcomes, associated with donor morbidity, should be always measured and reported in surgical cases where donor tissue is used. The final COS-TBPI contains seven outcomes (Table 6).

**Table 6.** Core outcome set for adult TBPI.

Outcome domain	Description
Tier 1: Critically important to all stakeholders – ALWAYS measure and report	

Voluntary movement	To include all active movement of the whole upper limb, shoulder, elbow, wrist and hand
Pain	The experience of pain including pain intensity, duration and frequency, and description (quality and interference)
Carrying out daily routine	Daily routine to include housework, taking care of plants indoors and outdoors, preparing meals (expanded at consensus meeting to include maintaining personal hygiene, personal appearance, dressing and ability to carry out routine at work and with hobbies)

Tier 2: Important domains but optional to measure and report	

Emotional distress	The emotional impact on life (including work, ADL and relationships), energy levels, mood and anxiety and depression
Ability to feel with the arm	To include the ability to feel and the ability to feel to protect
Ability to cope	The ability to cope
Access to appropriate treatment	Access to appropriate treatment

Surgery involving donor sites: To be measured and reported in surgical cases where donor sites used	
Loss of voluntary movement	In previously unaffected donor muscles not already denervated from original BPI
Worsening of pain or pins and needles	In the distribution of affected TBPI nerves or donor sites

ADL: activities of daily living; TBPI: traumatic brachial plexus injury.

## Discussion

Through a large international consensus study, including surgeons, patients and therapists from 21 countries, we have established a COS for TBPI. The core outcome domains are voluntary movement, carrying out a daily routine and pain. At least 85% of international patients, surgeons and therapist participants identified these outcomes as critically important. As a minimum, these three outcomes should be measured and reported in clinical care and research in adults with a TBPI. Two complications of surgery (‘loss of voluntary movement’ and ‘worsening of pain or pins and needles’) are complications specific to donor morbidity and additional outcomes to include in surgical studies or in patients undergoing surgery using donor sites. The COS-TBPI represents a minimum set of outcomes. Four further outcomes were included in Tier 2, which were critically important to some but not all stakeholders. While important, they are optional outcomes to be measured and reported. Additional outcomes can be added at the clinicians’ or researchers’ discretion. No other published COS for TBPI has been identified.

The findings from our consensus process highlighted differences between the outcomes reported in current TBPI studies and those prioritized as important by HCP and patient stakeholder groups. Strength is measured and reported in approximately 90% of studies including patients with a TBPI (Dy et al., 2015; Miller et al., 2021). However, strength as a single domain was not prioritized by the stakeholders in our study. It is plausible that participants voted for broader domains, which include strength. For example, to perform ‘voluntary movement’ and ‘carrying out daily routine’, an individual needs strength in their upper limb. Only 25% of TBPI studies measure and report voluntary movement (assessed by either goniometry or visual estimation of active movement) and pain (Dy et al., 2015; Miller et al., 2021), but both domains met the threshold for consensus in the current study. Finally, TBPI studies infrequently report ‘carrying out daily routine’ (Dy et al., 2015; Miller et al., 2021) but the COS-TBPI includes it. The inclusion of outcomes not frequently measured and reported in the literature highlight the value of including patients and other HCPs in the consensus process, ensuring outcomes are relevant to all stakeholders.

Although there was some overlap in outcome priorities by professionals and patients, there were also some notable differences. At the end of the consensus meetings, nine outcomes were prioritized by either patients and/or HCPs. Five outcomes were prioritized by both groups (voluntary movement, carrying out a daily routine, two pain outcomes and the complication, loss of voluntary movement); the remaining four were prioritized by one group only (Table 5). The ‘ability to feel with the arm’ and ‘fine hand movement’ reached consensus for inclusion in the COS by the HCPs only, whereas ‘access to appropriate treatment’ and ‘worsening of pain or pins and needles’ reached consensus in the patient-only group. Differences in patient and professional views are common in COS development and have been seen in other disease areas (Blazeby et al., 2015; Coulman et al., 2016; Fish et al., 2018; Potter et al., 2015). During the development of a COS for breast reconstruction and an oesophageal cancer core information set, patients rated the longer-term quality of life outcomes more highly than professionals did (Blazeby et al., 2015; Potter et al., 2015). In contrast, patients in our consensus process did not rate quality of life or emotional well-being outcomes highly. This may be because the patient participants are generally young men of working age, a very different demographic to the patient participants in the other studies, which focused on cancer and included older participants.

The COMBINE study has several strengths. The methods adhered to international recommendations (Kirkham et al., 2017b) and were defined a priori in a protocol (Miller et al., 2019). The inclusion of both patients and HCPs at every stage ensured the outcomes in the final COS were representative of their shared priorities. The views of different stakeholder groups were represented equally, despite a difference in the number of participants. The comprehensive and rigorous long-listing process combining data from a systematic review and patient interviews ensured that outcomes across all COMET domains were considered in the consensus process.

The present study has some limitations. Retention of each stakeholder group over the three Delphi rounds was in the range of 61%–86%, with surgeons having the highest attrition rates. There is no recommendation on acceptable response rates for a Delphi. Loss of participants could mean that people with minority opinions drop out, leading to an overestimation of consensus, thus affecting the validity of the results (Hasson et al., 2000). The COMBINE Delphi was conducted over the summer period and during the COVID-19 pandemic in 2021, which may have affected retention rates for the HCPs.

Some stakeholders, including family and carers, were not included in the consensus process. These groups may have provided different perspectives. We included stakeholders from 21 countries; however, future research is needed to explore whether the current COS reflects the priorities of individuals with TBPI more widely particularly in non-English speaking populations. Furthermore, to ensure quality and consistency in measurement and reporting of outcomes, further work is needed to identify the best outcome measurement instruments for each outcome in the COS-TBPI. This will enhance its uptake and implementation.

In conclusion, we used an international consensus process to agree on a minimum set of core outcomes to be measured and reported when evaluating adult TBPI interventions. The COS-TBPI defines ‘what’ should be measured and not ‘how’ the outcomes are measured. Further work on outcome measurement for TBPI is needed to establish ‘how’ best to measure these outcomes. The COS-TBPI represents the consensus from an international group of patients, therapists and surgeons and should be used in future clinical studies and in routine care of adults with TBPI. Implementation of the COS-TBPI will enhance the relevance of study findings and treatments to patients, HCPs and researchers as well as enable consistent reporting and effective data synthesis in support of evidence-based healthcare for patients with TBPI.

## Supplemental Material

sj-pdf-1-jhs-10.1177_17531934231212973 - Supplemental material for Development of a core outcome set for traumatic brachial plexus injury Supplemental material, sj-pdf-1-jhs-10.1177_17531934231212973 for Development of a core outcome set for traumatic brachial plexus injury by Caroline Miller, Jane Cross, Dominic M. Power and Christina Jerosch-Herold in Journal of Hand Surgery (European Volume)

sj-pdf-2-jhs-10.1177_17531934231212973 - Supplemental material for Development of a core outcome set for traumatic brachial plexus injury Supplemental material, sj-pdf-2-jhs-10.1177_17531934231212973 for Development of a core outcome set for traumatic brachial plexus injury by Caroline Miller, Jane Cross, Dominic M. Power and Christina Jerosch-Herold in Journal of Hand Surgery (European Volume)

sj-pdf-3-jhs-10.1177_17531934231212973 - Supplemental material for Development of a core outcome set for traumatic brachial plexus injury Supplemental material, sj-pdf-3-jhs-10.1177_17531934231212973 for Development of a core outcome set for traumatic brachial plexus injury by Caroline Miller, Jane Cross, Dominic M. Power and Christina Jerosch-Herold in Journal of Hand Surgery (European Volume)

sj-pdf-4-jhs-10.1177_17531934231212973 - Supplemental material for Development of a core outcome set for traumatic brachial plexus injury Supplemental material, sj-pdf-4-jhs-10.1177_17531934231212973 for Development of a core outcome set for traumatic brachial plexus injury by Caroline Miller, Jane Cross, Dominic M. Power and Christina Jerosch-Herold in Journal of Hand Surgery (European Volume)

## Registration

This COMBINE project was pre-registered on the COMET Initiative database before commencement (https://www.comet-initiative.org/).
