# Supplementary material for: Development of a core outcome set for traumatic brachial plexus injury
Source: J Hand Surg Eur Vol. 2023 Nov 21;49(5):554–63. doi: 10.1177/17531934231212973 (PMC11044516; doi:10.1177/17531934231212973)
Supplement: sj-pdf-1-jhs-10.1177_17531934231212973 - Supplemental material for Development of a core outcome set for traumatic brachial plexus injury [file sj-pdf-1-jhs-10.1177_17531934231212973.pdf]

Table S1. Outcomes for voting at consensus meeting.

General outcomes

Outcomes	Outcomes
Voluntary movement	Appropriateness of treatment
Strength of the arm	The ability of the brachial plexus nerves to send signals to the skin and muscles of the arm
Carrying and lifting	Carrying out daily routine
Fine hand movement	Maintaining personal hygiene
Ability to feel with the arm	Putting on and taking off clothes
Ability to feel to protect the arm from injury	Ability to eat using the utensils/ hands
Pain intensity	Effect on relationship with or ability to care for children
Pain duration	Emotional distress
Pain description	Self -confidence
Overall health	Ability to cope
Access to treatment	Expectations of treatment

Complication Outcomes

Outcomes	Outcomes
Loss of voluntary movement	Failure of a surgical join of the nerve
Loss of assisted movement (passive)	Failure of a surgical join of an artery of a vein
Limited voluntary movement because of inability to co-ordinate muscles at the same time	Injury to an artery or vein resulting in bleeding where the operation takes place
Nerve forms a painful bundle of nerves (neuroma)	Development of a blood clot
Damage to other nerves during the surgery	Breathing problems
Worsening of existing pain or pins and needles	
