# Supplementary material for: Development of a core outcome set for traumatic brachial plexus injury
Source: J Hand Surg Eur Vol. 2023 Nov 21;49(5):554–63. doi: 10.1177/17531934231212973 (PMC11044516; doi:10.1177/17531934231212973)
Supplement: sj-pdf-2-jhs-10.1177_17531934231212973 - Supplemental material for Development of a core outcome set for traumatic brachial plexus injury [file sj-pdf-2-jhs-10.1177_17531934231212973.pdf]

Table S2. Long list of outcomes with domain categorisation.

**Domain name**	**Outcomes**
Physical signs (movement, strength and ability)	Voluntary movement of the arm
	Passive/ assisted movement of the arm
	Strength of muscles in the arm
	The physical appearance of the arm
	Reaching, pulling, pushing, turning or twisting with the arm
	Carrying and lifting objects
	Fine hand movement
Sensation and pain in the arm	Ability to feel with the arm
	Ability to feel in order to protect the arm from injury
	Sensation of heaviness in the arm
	Pins and needles or tingling in the arm
	Pain intensity

	Pain duration and frequency
	Description of the pain
	Pain when the arm is exposed to cold
Neurophysiology and structure of nervous system	The ability of the brachial plexus nerves to send signals to the skin and muscles in the arm.
	The structure of brachial plexus using MRI or other techniques
	A measure of the activity in the movement and sensation areas of the brain.
Activities of daily living and work	Carrying out a daily routine
	Maintaining personal hygiene
	Maintaining personal appearance
	Putting on and taking off clothes
	Transport needs
	Return to full duties at previous role in paid employment
	Return to or begin role in education
	Return to any other paid/non paid previous role

	Return to previous recreational activities
Social well-being	Effect on relationship with partner/ spouse
	Effect on relationship with and or ability to care for, children
	Effect on relationship with other family members
	Effect on relationships with friends and neighbours
	Effects on intimate relationships
Emotional well-being	Emotional distress
	Thoughts and beliefs
	Intentions and goals
	Addictive behaviours (e.g alcohol, medication drugs)
	Body Image
	Self-esteem and self-confidence
Sleep and overall health	Overall quality of sleep
	Overall health

Delivery of care	Patient satisfaction with health care received<br>Access to and quality of treatment<br>Appropriateness of treatment
Costs of care	Out of pocket costs to the patient for outpatient appointments and inpatient care<br>Costs to the patient from long term loss of individual/ family income<br>Costs to uninsured private paying patients, insurance or other third party payer (includes national health services) for all out-patient and in-patient care received for a brachial plexus injury including medication
Complications (muscle and bone)	Loss of voluntary (active) movement<br>Loss of assisted range of motion (stiffness)<br>Failure of the bone to unite following bone surgery<br>Bone uniting in the wrong position

Complications (nerve related)	Damage to other nerves during the surgery
	Worsening of existing pain / pins and needles
	Development of pain/ pins and needles in a new area of the body
	Increased sensitivity of the scar
Complications (problems with surgical joins and infection)	A nerve join results in a formation of bundle of painful nerves
	Failure of the surgical join of the nerves
	Failure of the surgical join of the artery/ vein
	Infection in the body part that was operated on
	Problems with wounds such as infection, failure to heal properly
Complications (bleeding and breathing problems)	Injury to an artery or vein resulting in bleeding where the operation takes place
	Bleeding from the wound
	Development of a blood clot
	Breathing problems
	Chest infection
