# Supplementary material for: Development of a core outcome set for traumatic brachial plexus injury
Source: J Hand Surg Eur Vol. 2023 Nov 21;49(5):554–63. doi: 10.1177/17531934231212973 (PMC11044516; doi:10.1177/17531934231212973)
Supplement: sj-pdf-3-jhs-10.1177_17531934231212973 - Supplemental material for Development of a core outcome set for traumatic brachial plexus injury [file sj-pdf-3-jhs-10.1177_17531934231212973.pdf]

Table S3. A) New outcomes added to round 2 Delphi.

Domain	New outcome proposed wording	Hover wording[1]
**Physical signs**	Muscle fatigue or endurance	The ability of the muscle to sustain force over time
	Development of musculoskeletal problems in other parts of the body	Development of other musculoskeletal problems in other parts of the body secondary to traumatic brachial plexus injury
**Emotional wellbeing**	Expectations of treatment	To include expectations of benefit and side effects of treatments
	Ability to cope	Coping strategies to include self-efficacy (an individual's belief and confidence that they can exert control over their life)
**Activities of daily living**	Ability to eat using utensils or hand	To include the manipulation of utensils (knife and fork, chopsticks, etc) or using their fingers to eat in a socially and culturally appropriate way
**Complications (muscle or bone)**	Limited voluntary movement because of the inability to co-ordinate muscles at the same time (co-contraction)	Co-contraction or poor muscle coordination leads to a lack of voluntary movement.

[1] When a participant **hovers** over an outcome in the Delphi, a more detailed explanation of the outcome appears in a text box by the outcome.

Table S3. B) Revised wording of seven outcomes from feedback in round 1 Delphi.

Domain	Existing outcome	Proposed wording for the outcome	Proposed new wording for 'hover' function[1]	Rationale
**Physical signs**	Strength of muscles in the arm	No change<br>Strength of muscles in the arm	The ability of the muscle to generate enough force to work against gravity or resistance.	Several participants suggested that fatigue should be a separate outcome, so we removed it from the 'strength' outcome and made a new outcome
**Sensation and pain in the arm**	Ability to feel with the arm	Ability to feel with the arm including the hands and fingers	No change<br>For example, the ability to feel touch, texture, shape, and weight	Some participants commented and suggested the hand and fingers should be included as the sensation was more important in the hand than in the arm
**Activities of Daily Living**	Return to previous recreational activities	No change<br>Return to previous recreational activities	Including sports, gardening, and hobbies	Two participants suggested sports needed to be included
	Transport needs	Transport needs, including driving cars and riding motorbikes and bicycles	The ability to use transport including driving cars, riding motorbikes and bicycles	A comment suggested that riding bicycles were particularly important in certain parts of the world where these injuries occur. It was decided to move 'ride a bicycle' into the main outcome

[1] When a participant ***hovers*** over an outcome in the Delphi, a more detailed explanation of the outcome appears in a text box by the outcome.

Domain	Existing outcome	Proposed wording for the outcome	Proposed new wording for hover[1] function	Rationale
**Emotional wellbeing**	Emotional distress	Emotional distress including anxiety, depression, post-traumatic stress	To also include low mood, suicidal thoughts, flashbacks, and nightmares	Many comments suggested that we should be measuring depression, anxiety and PTSD. These were always included in the hover, so we moved them to the outcome title
	Thoughts and beliefs	Thoughts and beliefs	Including acceptance of injury	Remove expectations of treatment, as becoming a new outcome
	Access to and quality of treatment	Access to (waiting times, distance from, ease of referral) and quality of treatment	Including distance to centres, waiting times, and information regarding treatment	Many comments that ease of access to specific centres was important and needed to be a main outcome, not only in explanation.

[1] When a participant ***hovers*** over an outcome in the Delphi, a more detailed explanation of the outcome appears in a text box by the outcome.
