# Supplementary material for: Development of a core outcome set for traumatic brachial plexus injury
Source: J Hand Surg Eur Vol. 2023 Nov 21;49(5):554–63. doi: 10.1177/17531934231212973 (PMC11044516; doi:10.1177/17531934231212973)
Supplement: sj-pdf-4-jhs-10.1177_17531934231212973 - Supplemental material for Development of a core outcome set for traumatic brachial plexus injury [file sj-pdf-4-jhs-10.1177_17531934231212973.pdf]

Table S4. Consensus meetings participants type by country.

Country of participant	Patients<br>n = 13	Clinicians<br>n = 25	Total<br>n = 38
United Kingdom	8	11	19
Australia	2	2	4
US	1	2	3
Canada	1	1	2
Sweden	1	5	6
Argentina	0	1	1
South Africa	0	1	1
Switzerland	0	1	1
Denmark	0	1	1
